# 4-Meth­oxy-*N*′-(2-methoxy­benzyl­idene)benzohydrazide

**DOI:** 10.1107/S1600536809019102

**Published:** 2009-05-23

**Authors:** Xinyou Zhang

**Affiliations:** a Department of Chemistry, Baicheng Normal College, Baicheng 137000, People’s Republic of China

## Abstract

In the title compound, C_16_H_16_N_2_O_3_, the two benzene rings are inclined to one another by 75.4 (2)°, and the mol­ecule adopts an *E* configuration about the C=N bond. In the crystal structure, symmetry-related mol­ecules are linked *via* inter­molecular N—H⋯O hydrogen bonds, forming chains running parallel to the *c* axis.

## Related literature

For related structures, see: Alhadi *et al.* (2008 ▶), Küçükgüzel *et al.* (2003 ▶); Mohd Lair *et al.* (2009*a* ▶,*b* ▶); Li *et al.* (2009 ▶); Zhang *et al.* (2009 ▶). For reference structural data, see: Allen *et al.* (1987 ▶).
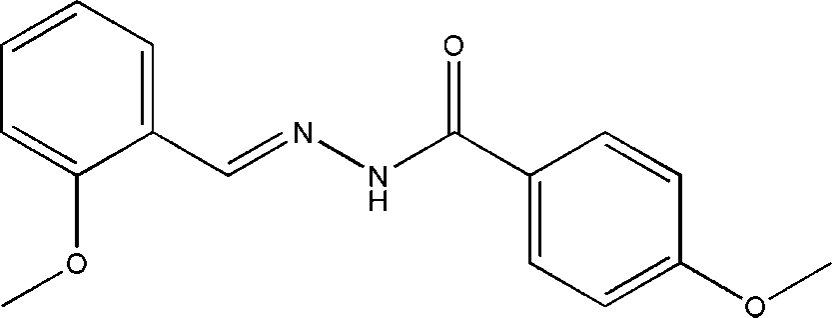


## Experimental

### 

#### Crystal data

C_16_H_16_N_2_O_3_
*M* *_r_* = 284.31
Monoclinic, 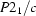

*a* = 12.705 (1) Å
*b* = 16.053 (2) Å
*c* = 7.718 (1) Å
β = 107.233 (2)°
*V* = 1503.5 (3) Å^3^
*Z* = 4
Mo *K*α radiation
μ = 0.09 mm^−1^
*T* = 298 K
0.20 × 0.20 × 0.18 mm

#### Data collection

Bruker SMART CCD area-detector diffractometer
Absorption correction: multi-scan (*SADABS*; Sheldrick, 1996 ▶) *T*_min_ = 0.983, *T*_max_ = 0.984
8570 measured reflections
3007 independent reflections
1574 reflections with *I* > 2σ(*I*)
*R*_int_ = 0.050

#### Refinement

*R*[*F* ^2^ > 2σ(*F* ^2^)] = 0.049
*wR*(*F* ^2^) = 0.123
*S* = 0.98
3007 reflections
195 parameters
1 restraint
H atoms treated by a mixture of independent and constrained refinement
Δρ_max_ = 0.13 e Å^−3^
Δρ_min_ = −0.19 e Å^−3^

### 

Data collection: *SMART* (Bruker, 2002 ▶); cell refinement: *SAINT* (Bruker, 2002 ▶); data reduction: *SAINT*; program(s) used to solve structure: *SHELXS97* (Sheldrick, 2008 ▶); program(s) used to refine structure: *SHELXL97* (Sheldrick, 2008 ▶); molecular graphics: *SHELXTL* (Sheldrick, 2008 ▶); software used to prepare material for publication: *SHELXL97*.

## Supplementary Material

Crystal structure: contains datablocks global, I. DOI: 10.1107/S1600536809019102/su2114sup1.cif

Structure factors: contains datablocks I. DOI: 10.1107/S1600536809019102/su2114Isup2.hkl

Additional supplementary materials:  crystallographic information; 3D view; checkCIF report

## Figures and Tables

**Table 1:** Hydrogen-bond geometry (Å, °)

*D*—H⋯*A*	*D*—H	H⋯*A*	*D*⋯*A*	*D*—H⋯*A*
N2—H2⋯O2^i^	0.898 (9)	1.985 (11)	2.859 (2)	164 (2)

Symmetry code: (i) 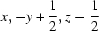.

**Fig. 1.**
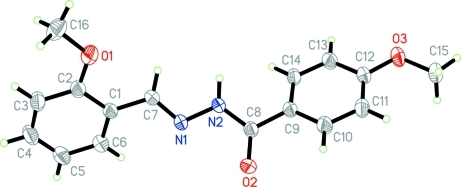
The molecular structure of the title compound, with displacement ellipsoids drawn at the 30% probabilty level.

**Fig. 2.**
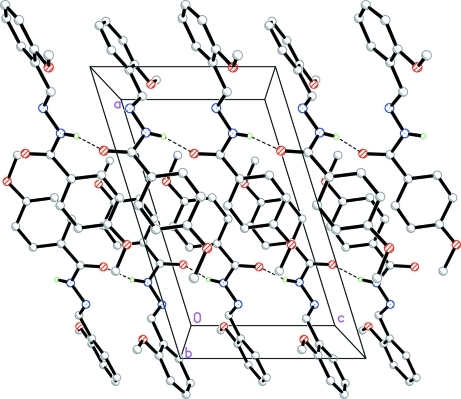
A view along the b axis of the crystal packing of the title compound, with hydrogen bonds shown as dashed lines (see Table 1 for details). H-atoms not involved in hydrogen bonding have been omitted for clarity.

## supplementary crystallographic information

### Comment

Hydrazone compounds are readily synthesized by the reaction of aldehydes with hydrazides. Recently, a large number of such compounds have been reported on (Alhadi *et al.*, 2008; Küçükgüzel *et al.*, 2003; Li *et al.*, 2009; Zhang *et al.*, 2009). In continuation of work in this area we report herein on the crystal structure of the title compound, a new hydrazone compound synthesized from the reaction of equimolar quantities of 2-methoxybenzaldehyde with 4-methoxybenzohydrazide in methanol.

The molecule structure of the title compound is illustrated in Fig. 1. The molecule adopts an *E* configuration about the C═N bond. The dihedral angle involving the two benzene rings is 75.4 (2)°. All the bond lengths are within normal values (Allen *et al.*, 1987) and are comparable with those observed in the similar compounds (Mohd Lair *et al.*, 2009*a*,b).

In the crystal structure of the compound, symmetry related molecules are linked through intermolecular N–H···O and N–H···N hydrogen bonds, forming chains running along the *c* axis (Table 1 and Fig. 2).

### Experimental

2-Methoxybenzaldehyde (1.0 mmol, 136.2 mg) and 4-methoxybenzohydrazide (1.0 mmol, 166.2 mg) were mixed in a methanol solution, and the mixture was refluxed for 1 h. Colorless block-shaped crystals of the title compound were formed by slow evaporation of the solution in air.

### Refinement

Atom H2 attached to N2 was located from a difference Fourier map and freely refined with *U*_iso_(H) restrained to 0.08 Å^2^. The C-bound H-atoms were included in calculated positions and refined as riding atoms: d(C–H) = 0.93–0.96 Å, with *U*_iso_(H) = 1.2*U*_eq_(C), or 1.5*U*_eq_(C_methyl_).

### Crystal data

**Table:**

C_16_H_16_N_2_O_3_	*F*(000) = 600
*M**_r_* = 284.31	*D*_x_ = 1.256 Mg m^−^^3^
Monoclinic, *P*2_1_/*c*	Mo *K*α radiation, λ = 0.71073 Å
Hall symbol: -P 2ybc	Cell parameters from 1023 reflections
*a* = 12.705 (1) Å	θ = 2.5–24.6°
*b* = 16.053 (2) Å	µ = 0.09 mm^−^^1^
*c* = 7.718 (1) Å	*T* = 298 K
β = 107.233 (2)°	Block, colorless
*V* = 1503.5 (3) Å^3^	0.20 × 0.20 × 0.18 mm
*Z* = 4	

### Data collection

**Table:**

Bruker SMART CCD area-detector diffractometer	3007 independent reflections
Radiation source: fine-focus sealed tube	1574 reflections with *I* > 2σ(*I*)
graphite	*R*_int_ = 0.050
ω scans	θ_max_ = 26.2°, θ_min_ = 1.7°
Absorption correction: multi-scan (*SADABS*; Sheldrick, 1996)	*h* = −15→13
*T*_min_ = 0.983, *T*_max_ = 0.984	*k* = −19→17
8570 measured reflections	*l* = −9→9

### Refinement

**Table:**

Refinement on *F*^2^	Primary atom site location: structure-invariant direct methods
Least-squares matrix: full	Secondary atom site location: difference Fourier map
*R*[*F*^2^ > 2σ(*F*^2^)] = 0.049	Hydrogen site location: inferred from neighbouring sites
*wR*(*F*^2^) = 0.123	H atoms treated by a mixture of independent and constrained refinement
*S* = 0.98	*w* = 1/[σ^2^(*F*_o_^2^) + (0.0515*P*)^2^] where *P* = (*F*_o_^2^ + 2*F*_c_^2^)/3
3007 reflections	(Δ/σ)_max_ < 0.001
195 parameters	Δρ_max_ = 0.13 e Å^−^^3^
1 restraint	Δρ_min_ = −0.18 e Å^−^^3^

### Special details

**Table:**

Geometry. All e.s.d.'s (except the e.s.d. in the dihedral angle between two l.s. planes) are estimated using the full covariance matrix. The cell e.s.d.'s are taken into account individually in the estimation of e.s.d.'s in distances, angles and torsion angles; correlations between e.s.d.'s in cell parameters are only used when they are defined by crystal symmetry. An approximate (isotropic) treatment of cell e.s.d.'s is used for estimating e.s.d.'s involving l.s. planes.
Refinement. Refinement of *F*^2^ against ALL reflections. The weighted *R*-factor *wR* and goodness of fit *S* are based on *F*^2^, conventional *R*-factors *R* are based on *F*, with *F* set to zero for negative *F*^2^. The threshold expression of *F*^2^ > σ(*F*^2^) is used only for calculating *R*-factors(gt) *etc*. and is not relevant to the choice of reflections for refinement. *R*-factors based on *F*^2^ are statistically about twice as large as those based on *F*, and *R*- factors based on ALL data will be even larger.

### Fractional atomic coordinates and isotropic or equivalent isotropic displacement parameters (Å^2^)

**Table:**

	*x*	*y*	*z*	*U*_iso_*/*U*_eq_	
N1	0.11626 (13)	0.25479 (11)	0.8800 (2)	0.0459 (5)	
N2	0.20751 (14)	0.23123 (11)	0.8290 (2)	0.0464 (5)	
O1	−0.00123 (13)	0.47737 (10)	0.7280 (2)	0.0698 (5)	
O2	0.27806 (12)	0.16021 (9)	1.0915 (2)	0.0572 (4)	
O3	0.62405 (13)	0.06696 (12)	0.6746 (2)	0.0832 (6)	
C1	−0.03802 (17)	0.34548 (14)	0.8236 (3)	0.0484 (6)	
C2	−0.07099 (18)	0.42835 (15)	0.7881 (3)	0.0532 (6)	
C3	−0.1678 (2)	0.45577 (18)	0.8150 (3)	0.0689 (8)	
H3	−0.1900	0.5108	0.7906	0.083*	
C4	−0.2316 (2)	0.4011 (2)	0.8783 (3)	0.0777 (8)	
H4	−0.2970	0.4199	0.8958	0.093*	
C5	−0.2007 (2)	0.3201 (2)	0.9158 (4)	0.0782 (8)	
H5	−0.2440	0.2838	0.9593	0.094*	
C6	−0.10359 (18)	0.29291 (16)	0.8878 (3)	0.0629 (7)	
H6	−0.0821	0.2378	0.9130	0.075*	
C7	0.06430 (17)	0.31710 (14)	0.7935 (3)	0.0477 (6)	
H7	0.0918	0.3448	0.7103	0.057*	
C8	0.28308 (17)	0.18075 (13)	0.9401 (3)	0.0456 (5)	
C9	0.37276 (17)	0.15184 (13)	0.8685 (3)	0.0467 (6)	
C10	0.47380 (19)	0.13301 (16)	0.9881 (3)	0.0693 (7)	
H10	0.4844	0.1393	1.1119	0.083*	
C11	0.5597 (2)	0.10505 (18)	0.9289 (4)	0.0772 (8)	
H11	0.6276	0.0932	1.0121	0.093*	
C12	0.54480 (19)	0.09484 (15)	0.7470 (3)	0.0598 (7)	
C13	0.44375 (19)	0.11214 (15)	0.6249 (3)	0.0620 (7)	
H13	0.4330	0.1045	0.5014	0.074*	
C14	0.35890 (17)	0.14068 (13)	0.6849 (3)	0.0527 (6)	
H14	0.2912	0.1527	0.6013	0.063*	
C15	0.7272 (2)	0.04266 (18)	0.7970 (4)	0.0888 (9)	
H15A	0.7609	0.0897	0.8689	0.133*	
H15B	0.7745	0.0226	0.7295	0.133*	
H15C	0.7157	−0.0007	0.8750	0.133*	
C16	−0.0288 (2)	0.56282 (15)	0.6910 (4)	0.0780 (8)	
H16A	−0.0998	0.5670	0.6022	0.117*	
H16B	0.0255	0.5890	0.6454	0.117*	
H16C	−0.0309	0.5902	0.8006	0.117*	
H2	0.2197 (19)	0.2603 (13)	0.737 (2)	0.080*	

### Atomic displacement parameters (Å^2^)

**Table:**

	*U*^11^	*U*^22^	*U*^33^	*U*^12^	*U*^13^	*U*^23^
N1	0.0431 (10)	0.0511 (12)	0.0482 (11)	0.0074 (9)	0.0208 (9)	0.0010 (9)
N2	0.0464 (10)	0.0510 (12)	0.0481 (12)	0.0108 (9)	0.0236 (9)	0.0054 (9)
O1	0.0741 (11)	0.0536 (11)	0.0878 (13)	0.0155 (9)	0.0333 (10)	0.0116 (9)
O2	0.0637 (10)	0.0662 (10)	0.0492 (9)	0.0145 (8)	0.0284 (8)	0.0100 (8)
O3	0.0635 (11)	0.1180 (15)	0.0804 (13)	0.0380 (10)	0.0401 (11)	0.0142 (11)
C1	0.0489 (13)	0.0565 (15)	0.0416 (13)	0.0076 (11)	0.0162 (11)	−0.0018 (11)
C2	0.0526 (14)	0.0630 (16)	0.0447 (14)	0.0127 (13)	0.0154 (11)	−0.0016 (11)
C3	0.0665 (17)	0.085 (2)	0.0557 (16)	0.0325 (15)	0.0195 (14)	0.0003 (13)
C4	0.0543 (16)	0.121 (3)	0.0627 (18)	0.0267 (17)	0.0252 (14)	0.0017 (17)
C5	0.0587 (16)	0.106 (2)	0.080 (2)	0.0090 (16)	0.0360 (15)	0.0130 (17)
C6	0.0578 (15)	0.0715 (17)	0.0648 (17)	0.0077 (13)	0.0266 (13)	0.0044 (13)
C7	0.0484 (13)	0.0530 (14)	0.0458 (13)	0.0035 (11)	0.0202 (11)	0.0012 (11)
C8	0.0450 (13)	0.0454 (13)	0.0493 (14)	0.0025 (11)	0.0182 (11)	−0.0011 (11)
C9	0.0451 (13)	0.0513 (14)	0.0454 (13)	0.0055 (10)	0.0162 (11)	−0.0009 (10)
C10	0.0563 (15)	0.106 (2)	0.0462 (15)	0.0222 (15)	0.0168 (13)	0.0041 (14)
C11	0.0512 (15)	0.120 (2)	0.0616 (18)	0.0299 (15)	0.0180 (13)	0.0122 (16)
C12	0.0540 (15)	0.0704 (17)	0.0627 (17)	0.0178 (12)	0.0292 (14)	0.0071 (13)
C13	0.0608 (15)	0.0799 (18)	0.0499 (15)	0.0157 (13)	0.0235 (13)	−0.0013 (12)
C14	0.0433 (13)	0.0639 (15)	0.0520 (15)	0.0104 (11)	0.0157 (11)	−0.0005 (12)
C15	0.0634 (18)	0.112 (2)	0.102 (2)	0.0400 (16)	0.0407 (17)	0.0286 (18)
C16	0.094 (2)	0.0503 (17)	0.085 (2)	0.0136 (14)	0.0189 (17)	0.0054 (14)

### Geometric parameters (Å, °)

**Table:**

N1—C7	1.273 (2)	C6—H6	0.9300
N1—N2	1.383 (2)	C7—H7	0.9300
N2—C8	1.351 (3)	C8—C9	1.481 (3)
N2—H2	0.898 (9)	C9—C10	1.375 (3)
O1—C2	1.365 (3)	C9—C14	1.387 (3)
O1—C16	1.424 (3)	C10—C11	1.378 (3)
O2—C8	1.234 (2)	C10—H10	0.9300
O3—C12	1.364 (2)	C11—C12	1.369 (3)
O3—C15	1.424 (3)	C11—H11	0.9300
C1—C6	1.377 (3)	C12—C13	1.377 (3)
C1—C2	1.397 (3)	C13—C14	1.372 (3)
C1—C7	1.460 (3)	C13—H13	0.9300
C2—C3	1.379 (3)	C14—H14	0.9300
C3—C4	1.378 (3)	C15—H15A	0.9600
C3—H3	0.9300	C15—H15B	0.9600
C4—C5	1.366 (4)	C15—H15C	0.9600
C4—H4	0.9300	C16—H16A	0.9600
C5—C6	1.384 (3)	C16—H16B	0.9600
C5—H5	0.9300	C16—H16C	0.9600
			
C7—N1—N2	114.46 (17)	C10—C9—C14	117.94 (19)
C8—N2—N1	118.77 (17)	C10—C9—C8	119.13 (19)
C8—N2—H2	123.7 (15)	C14—C9—C8	122.9 (2)
N1—N2—H2	115.9 (15)	C9—C10—C11	121.5 (2)
C2—O1—C16	118.51 (18)	C9—C10—H10	119.2
C12—O3—C15	117.7 (2)	C11—C10—H10	119.2
C6—C1—C2	118.4 (2)	C12—C11—C10	119.8 (2)
C6—C1—C7	121.9 (2)	C12—C11—H11	120.1
C2—C1—C7	119.7 (2)	C10—C11—H11	120.1
O1—C2—C3	124.4 (2)	O3—C12—C11	124.4 (2)
O1—C2—C1	115.38 (19)	O3—C12—C13	115.9 (2)
C3—C2—C1	120.2 (2)	C11—C12—C13	119.7 (2)
C4—C3—C2	119.6 (3)	C14—C13—C12	120.2 (2)
C4—C3—H3	120.2	C14—C13—H13	119.9
C2—C3—H3	120.2	C12—C13—H13	119.9
C5—C4—C3	121.3 (2)	C13—C14—C9	120.9 (2)
C5—C4—H4	119.4	C13—C14—H14	119.6
C3—C4—H4	119.4	C9—C14—H14	119.6
C4—C5—C6	118.7 (3)	O3—C15—H15A	109.5
C4—C5—H5	120.6	O3—C15—H15B	109.5
C6—C5—H5	120.6	H15A—C15—H15B	109.5
C1—C6—C5	121.7 (2)	O3—C15—H15C	109.5
C1—C6—H6	119.2	H15A—C15—H15C	109.5
C5—C6—H6	119.2	H15B—C15—H15C	109.5
N1—C7—C1	120.65 (19)	O1—C16—H16A	109.5
N1—C7—H7	119.7	O1—C16—H16B	109.5
C1—C7—H7	119.7	H16A—C16—H16B	109.5
O2—C8—N2	122.35 (19)	O1—C16—H16C	109.5
O2—C8—C9	122.1 (2)	H16A—C16—H16C	109.5
N2—C8—C9	115.56 (19)	H16B—C16—H16C	109.5

### Hydrogen-bond geometry (Å, °)

**Table:**

*D*—H···*A*	*D*—H	H···*A*	*D*···*A*	*D*—H···*A*
N2—H2···O2^i^	0.90 (1)	1.99 (1)	2.859 (2)	164 (2)

Symmetry codes: (i) *x*, −*y*+1/2, *z*−1/2.
